# Taiwan Biobank: making cross-database convergence possible in the Big Data era

**DOI:** 10.1093/gigascience/gix110

**Published:** 2017-11-15

**Authors:** Jui-Chu Lin, Chien-Te Fan, Chia-Cheng Liao, Yao-Sheng Chen

**Affiliations:** 1 National Taiwan University of Science and Technology, No. 43, Keelung Rd., Sec. 4, Dayan District, Taipei, Taiwan 10607 (ROC); 2 Institute of Law for Science and Technology National Tsing Hua University, No. 101, Sec. 2, Kuang-Fu Rd., Hsinchu, Taiwan 30013 (ROC); 3 Saint Island International Patent & Law Offices, 11F-1, No. 248, Sec. 3, Nanking E. Rd., Taipei, Taiwan 10595

**Keywords:** EMR, biobank, precision medicine, linkage

## Abstract

The Taiwan Biobank (TWB) is a biomedical research database of biopsy data from 200 000 participants. Access to this database has been granted to research communities taking part in the development of precision medicines; however, this has raised issues surrounding TWB’s access to electronic medical records (EMRs). The Personal Data Protection Act of Taiwan restricts access to EMRs for purposes not covered by patients’ original consent. This commentary explores possible legal solutions to help ensure that the access TWB has to EMR abides with legal obligations, and with governance frameworks associated with ethical, legal, and social implications. We suggest utilizing “hash function” algorithms to create nonretrospective, anonymized data for the purpose of cross-transmission and/or linkage with EMR.

## Introduction

Since the completion of the Human Genome Project in 2003 [1], the biomedical industry has sought to demystify the causal links between a person's genes, the surrounding environment, and disease. Now, the adventure that began in the genomic era has entered the era of “Big Data.” Links are now being made between the biobanks that store genetic data and health databases that store electronic medical records (EMR) to boost biomedical research and to bring us closer to precision medicine. The Precision Medicine Initiative Cohort Program (PMI-CP) in the United States, the 100 000 Genomes Project in the United Kingdom, and the China Kadoorie Biobank in China are some ambitious national projects that exemplify this trend [2, 3].

**Figure 1:**
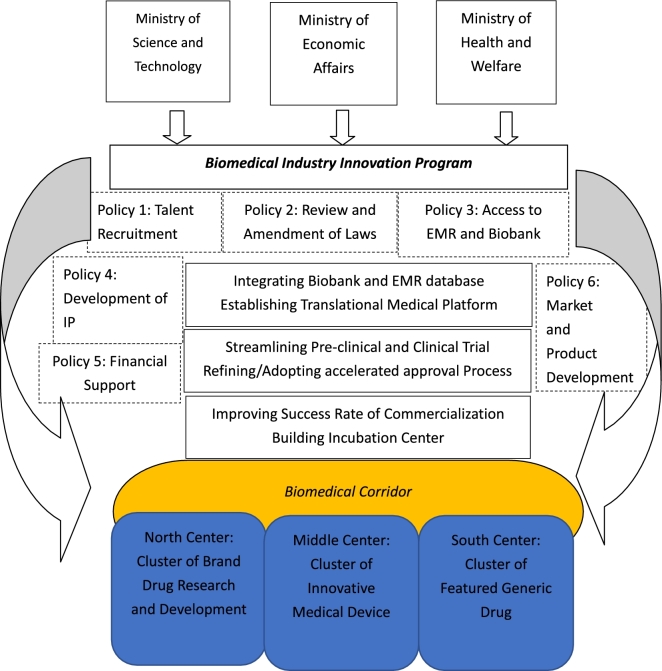
Flowchart of the BIIP. There are 6 featured policy aspects of the BIIP: talent recruitment, amendments of the law, increased access to electronic medical records and the Taiwan Biobank, development of intellectual property rights, providing financial support, and market and product development. By implementing these 6 policies, the BIIP aims to establish a biomedical corridor that extends from north to south, with 3 major biomedical centers that will be hubs for the development of Taiwan's biomedical industry. Under such a framework, a translational medical platform based on the integration of EMR and the Biobank will be the backbone for streamlining biomedical research. IP: intellectual property.

Taiwan has one of the most complete health-related databases in the world, with records covering up to 99% of the population of 23.5 million people [4]. To keep pace with the Big Data revolution, a Biomedical Industry Innovation Program (BIIP) was launched to promote a national translational medical research platform that would facilitate development in the biomedical industry and improve Taiwanese public health [5]. When planning this initiative, consideration was required for the strict patient protections afforded under the Personal Data Protection Act (PDPA), and the difficulty of accessing EMRs stored in the National Health Insurance Database (NHID), both of which could limit the effectiveness of the BIIP. Recently, the Ministry of Health and Welfare (MOHW) of Taiwan revised its practical guideline for the research-oriented use of the NHID. A major reason for this was to enable convergence between the TW Biobank (TWB) and the NHID (see Fig. 1).

### The challenge: the impact of the PDPA

Under the PDPA, any use of personal data, unless otherwise provided under the PDPA, shall be subject to autonomous, informed consent by the person with entitlement to those data. However, the health-related data in the NHID are collected for research purposes based on the National Health Insurance Law; that is, without prior informed consent. Therefore, harmonization between these 2 laws is critical in implementing the BIIP.

In 2013, a human rights group sued the National Health Insurance Bureau (NHIB) of the MOHW [6]. It was alleged that the PDPA was violated by granting access to NHID data for academic use—even though the data are encrypted and only accessible within a restricted area by authorized personnel. It was also alleged that the NHIB illegally transferred NHID data to a publicly accessible National Health Insurance Research Database (NHIRD) without prior informed consent.

After some deliberation, the Administrative Supreme Court of Taiwan sided with the NHIB, holding that although the proposed use of the data was without prior personal informed consent, it was justifiable under exemption clauses in the PDPA (see Table 1), and permissible because it was related to the NHIB’s statutory mandate. The court reasoned that even though the data were not anonymized, the measures taken by NHIB were sufficient to minimize the risk of undue exposure. The court also emphasized that a personal entitlement to privacy protection is not an absolute legal interest by nature and, when necessary, may be waived in cases of comparatively bigger public interest.

Despite the court's ruling, uncertainty remains. While human rights groups continue to question the legitimacy of academic access to NHID data, it also remains to be clarified whether a for-profit organization, such as a pharmaceutical company, might be granted similar access. Without resolving these PDPA issues, it is challenging for TW Biobank to develop a legally admissible cross-database convergence scheme to assist the MOHW in complementing the goals of the BIIP.

### The opportunity: the preliminary broad consent arrangement of the TW Biobank makes cross-database convergence possible

TW Biobank is a national biobank created under the supervision of the MOHW. It aims to collect data from 200 000 healthy participants and 100 000 individuals with 12 specific diseases to form the largest population-based biobank in Taiwan [7]. The fundamental goal of the TW Biobank is to facilitate cross-database linkage; therefore, each participant who has contributed data gave informed consent during the recruitment process, including for any future cross-database linkage, and for personal data collected in the NHID. Theoretically, the informed consent obtained by TW Biobank satisfies the exemption clause stipulated under Article 6, Paragraph 1, Subparagraph 6 of the PDPA (see below). The consent obtained during the recruitment phase of the development of TW Biobank is likely to already meet the criteria of the “prior personal consent with autonomy” exemption.

Personal information, such as details of medical treatment, genetics, sexual activity, health examinations, and criminal records, is sensitive in nature. Article 6 of the PDPA states that such information, in general, shall not be collected, processed, or used, except when:

1. in accordance with the law;
2. it is necessary for a government agency to perform its legal duties, or for a nongovernment agency to fulfill its legal obligations, and proper security measures are adopted prior or subsequent to such collection, processing, or use;
3. the Party has made public such information by himself, or when the information concerned has been publicized legally;
4. it is necessary to perform statistical or other academic research, and a government agency or an academic research institution collects, processes, or uses personal information for the purpose of medical treatment, public health, or crime prevention; the information may not lead to the identification of a specific person after its processing by the provider, or from the disclosure by the collector;
5. it is necessary to assist a government agency in performing its legal duties or a nongovernment agency in fulfilling its legal obligations, and proper security measures are adopted prior or subsequent to such collection, processing, or use;
6. the Party has consented in writing, unless such consent exceeds the necessary scope of the specific purpose; the collection, processing, or use merely with the consent of the Party is prohibited by other statutes; or such consent is against the Party's will.

Certainly, informed consent by itself is not a substitute for full compliance with ethical, legal, and social implications. This is especially true when the informed consent obtained by TWB is a broad consent; i.e., the consent has been obtained “for unspecified future research.” Besides, to facilitate the use of the Biobank, tissues and data collected may not remain unlinked. Thus, subject to Taiwan's 2010 Human Biobank Management Act (HBMA), participants have been asked to grant TWB the privilege to maintain the “irretrievability” of related data under the governance of the ethical code of “re-contact” and the continuous supervision of the Ethical Governance Committee (EGC). Furthermore, TWB may not release its collection without the approval of the EGC. To date, under this enhanced governance framework, more than 80 000 participants have been recruited, and no queries have been raised about the legitimacy of TWB’s practices.

However, it has been argued that until the PDPA is revised, its exemption clause should not be applicable to the broad consent practiced by TWB. While we believe that this would counter participants’ altruism and autonomy, concerns over the social legitimacy behind the argument cannot be ignored. Therefore, we suggest that TWB adopts an additional “hash function” to protect participants’ privacy.

**Figure 2:**
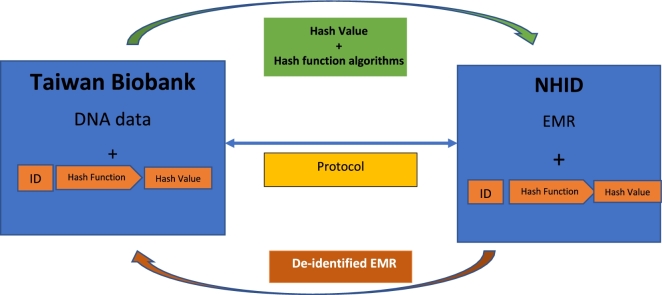
Flowchart describing the hash function framework. One of the key features of hash function algorithms is that they transform identifiable personal data into a unique 128-character code. The Taiwan Biobank will develop and enter into a protocol with the NHID and adopt a hash function framework. When it is necessary to access EMRs in the NHID, all identifiable data processed will be replaced by hash values returned by a hash function, so access to EMRs will proceed in an anonymous manner.

Biobanks such as the Vanderbilt DNA databank [8] have adopted hash functions that have proved useful in linking DNA data with health data in an anonymous fashion. Replacing the participant's ID with a hash value returned by a hash function further ensures that the participant’s identity cannot be regenerated from the same hash output (Fig. 2).

With sound broad consent from the participant for possible database linkage, continuing supervision by the EGC, and an additional hash function to bridge the gap between the HBMA and PDPA, it is possible for links between the TWB and NHID to survive the strict scrutiny of patient and human rights groups concerning PDPA compliance.

## Conclusion

In the Big Data era, it is not possible to achieve precision medicine without converging databases of genetic, environmental, and EMR information. However, the rigid protection of patient privacy is an obstacle for biobanks’ access to health databases. We believe a consent-based approach will help to ease concerns over violations of the PDPA and make the NHID accessible for research purposes. Further, with more and more national biobanks such as PMI-CP and the UK’s 100 000 Genomes Project being established, there is an increasing need for unified regional or international standards to ensure the interoperability of EMR. When implementing BIIP, the MOHW could use the TWB as an exemplar to standardize the procedure for accessing EMR in Taiwan and pave the way for Taiwan to be more active in global biobank networks.

## Abbreviations

BIIP: Biomedical Industry Innovation Program; EGC: Ethical Governance Committee; EMR: electronic medical records; HBMA: Human Biobank Management Act; MOHW: Ministry of Health and Welfare; NHIB: National Health Insurance Bureau; NHID: National Health Insurance Database; NHIRD: National Health Insurance Research Database; PDPA: Personal Data Protection Act; TWB: Taiwan Biobank.

**Table 1:** Article 6 of the PDPA

Personal information of medical records, medical treatment, genetic information, sexual life, health examination, and criminal records should not be collected, processed, or used. However, the following situations are not subject to the limits set in the preceding sentence: when in accordance with law; when it is necessary for a government agency to perform its legal duties or for a nongovernment agency to fulfill its legal obligation, and proper security measures are adopted prior or subsequent to such collection, processing, or use; when the Party has made public such information by himself, or when the information concerned has been publicized legally; where it is necessary to perform statistical or other academic research, a government agency or an academic research institution collects, processes, or uses personal information for the purpose of medical treatment, public health, or crime prevention; the information may not lead to the identification of a specific person after its processing by the provider, or from the disclosure by the collector; where it is necessary to assist a government agency in performing its legal duties or a nongovernment agency in fulfilling its legal obligations, and proper security measures are adopted prior or subsequent to such collection, processing, or use; where the Party has consented in writing, unless such consent exceeds the necessary scope of the specific purpose; the collection, processing or use merely with the consent of the Party is prohibited by other statutes; or such consent is against the Party's will.

Personal information like medical records, medical treatment, genetic information, sexual life, health examination, and criminal records, etc., is sensitive in nature. Article 6 of PDPA provides that such information, in general, shall not be collected, processed, or used. Article 6 of PDPA enlists 6 exceptions to this restriction.

## Supplementary Material

GIGA-D-17-00249_Original-Submission.pdf

GIGA-D-17-00249_Revision-1.pdf

Response-to-Reviewer-Comments_Original-Submission.pdf

Reviewer-1-Report-(Original-Submission) -- Ruth Stirton 13 Oct 2017 Reviewed
