# Supplementary material for: Taiwan Biobank: making cross-database convergence possible in the Big Data era
Source: Gigascience. 2017 Nov 15;7(1):gix110. doi: 10.1093/gigascience/gix110 (PMC5774504; doi:10.1093/gigascience/gix110)

GigaScience

# Making Possible the Cross Database Convergence of Taiwan Biobank in Big Data Era

--Manuscript Draft--

| | |
|---|---|
| Manuscript Number: | GIGA-D-17-00249 |
| Full Title: | Making Possible the Cross Database Convergence of Taiwan Biobank in Big Data Era |
| Article Type: | Commentary |
| Funding Information: | |
| Abstract: | Taiwan Biobank (TW Biobank) is a biopsy based database of 200,000 participants that provides data for biomedical research. While it has started granting access to research communities taking part in precision medicine development, the issues of TW Biobank's access to electronic medical records ("EMR") occurred. The Personal Data Protection Act of Taiwan imposes severe restrictions on access of EMR for purpose not covered by patient's original consent. This commentary explores possible legal solutions to make TW biobank's access of EMR abide with both legal and ethical obligations provided under Ethical, Legal & Social Implication governance frameworks. We suggest utilizing "Hash Function" algorithms to create non-retrospective data to achieve de-identification for the purpose of cross transmission and/or linkage for access of EMR. |
| Corresponding Author: | Jui-Chu Lin<br><br>TAIWAN |
| Corresponding Author Secondary Information: | |
| Corresponding Author's Institution: | |
| Corresponding Author's Secondary Institution: | |
| First Author: | Jui-Chu Lin |
| First Author Secondary Information: | |
| Order of Authors: | Jui-Chu Lin |
| | Chien-Te Fan |
| | Chia-Cheng Liao |
| | Yao-Sheng Chen |
| Order of Authors Secondary Information: | |
| Opposed Reviewers: | |
| Additional Information: | |
| **Question** | **Response** |
| Are you submitting this manuscript to a special series or article collection? | No |
| **Experimental design and statistics**<br><br>Full details of the experimental design and statistical methods used should be given in the Methods section, as detailed in our Minimum Standards Reporting Checklist. Information essential to interpreting the data presented should be made available in the figure legends. | Yes |

Powered by Editorial Manager® and ProduXion Manager® from Aries Systems Corporation

| | |
|---|---|
| Have you included all the information requested in your manuscript? | |
| **Resources**<br><br>A description of all resources used, including antibodies, cell lines, animals and software tools, with enough information to allow them to be uniquely identified, should be included in the Methods section. Authors are strongly encouraged to cite Research Resource Identifiers (RRIDs) for antibodies, model organisms and tools, where possible.<br><br>Have you included the information requested as detailed in our Minimum Standards Reporting Checklist? | Yes |
| **Availability of data and materials**<br><br>All datasets and code on which the conclusions of the paper rely must be either included in your submission or deposited in publicly available repositories (where available and ethically appropriate), referencing such data using a unique identifier in the references and in the "Availability of Data and Materials" section of your manuscript.<br><br>Have you have met the above requirement as detailed in our Minimum Standards Reporting Checklist? | Yes |

Manuscript Click here to download Manuscript Manuscript.doc

# Making Possible the Cross Database Convergence of Taiwan Biobank in Big Data Era

**Jui-Chu Lin[a, b,*], Chien-Te Fan[c], Chia-Cheng Liao[d], Yao-Sheng Chen[b]**

a. College of Humanities and Social Sciences, National Taiwan University of Science and Technology, Taipei 10607, Taiwan

b. Law and Technology Innovation Center, National Taiwan University of Science and Technology, Taipei 10607, Taiwan

c. Institute of Law for Science & Technology, National Tsing Hua University, Hsinchu 30013, Taiwan

d. Saint Island International Patent & Law Offices, Taipei 10595, Taiwan

***Corresponding author:**

**Jui-Chu Lin**

Director, Law and Technology Innovation Center

Distinguished professor, College of Humanities and Social Sciences

National Taiwan University of Science and Technology, Taipei, 10607, Taiwan.

No.43, Keelung Rd., Sec.4, Da'an Dist., Taipei City 10607, Taiwan

Tel: 886-2-2737-6967;

FAX: 886-2-2737-6288;

E-mail: 1030@mail.ntust.edu.tw

**Abstract**

Taiwan Biobank (TW Biobank) is a biopsy based database of 200,000 participants that provides data for biomedical research. While it has started granting access to research communities taking part in precision medicine development, the issues of TW Biobank's access to electronic medical records ("EMR") occurred. The Personal Data Protection Act of Taiwan imposes severe restrictions on access of EMR for purpose not covered by patient's original consent. This commentary explores possible legal solutions to make TW biobank's access of EMR abide with both legal and ethical obligations provided under Ethical, Legal & Social Implication governance frameworks. We suggest utilizing "Hash Function" algorithms to create non-retrospective data to achieve de-identification for the purpose of cross transmission and/or linkage for access of EMR.

## 1. Background

Since the completion of the human genome project in 2003 [1], the biomedical industry has embarked on a journey to demystify the causal links between one's genes, the surrounding environment, and disease. Now, the adventure that began in the genome era has entered the "Big Data" era. The linkage between biobanks that store genetic data and health databases that store electronic medical record (EMR) has been taken as a catalyst to boost biomedical research and to bring us a step closer to precision medicine. The PMI-CP in the U.S., the 100,000 Genomes Project from Genomics England in the U.K, and the China Kadoorie Biobank in China are some of the ambitious national projects exemplifying said trend [2, 3].

Taiwan has established one of the most complete health related databases in the world, with coverage up to 99% of the 23.5 million population [4]. To keep pace with the "Big Data" revolution, a Biomedical Industry Innovation Program ("BIIP") was promulgated, aiming to promote a national translational medical research platform, so as to facilitate biomedical industry development and improve public health in Taiwan [5] When so planning, due to the strict patient protections under the Personal Data Protection Act (PDPA), the accessibility of EMR stored in National Health Insurance Database ("NHID") is very difficult, which may limit and be necessary for BIIP

implementation. Recently, the Ministry of Health and Welfare ("MOHW") of Taiwan has revised its practical guideline for research oriented use of NHID. One of the main reasons has been the enablement of convergence between Taiwan Biobank and the NHID (See Figure. 1).

## 2. The Challenge: The Impact of the PDPA

Under PDPA, any use of personal data, unless otherwise provided under the PDPA, shall be subject to autonomous, informed consent by the person with entitlement to said data. However, NHID's health related data are collected based on the National Health Insurance Law, without prior informed consent for research purposes as required by PDPA. Therefore the harmonization between two laws become critical in BIIP implementation.

In 2013, the National Health Insurance Bureau ("NHIB") of MOHW was sued by a human right group [6]. It was alleged that NHIB's granting access for academic use of the NHID data, even in an encrypted manner and available in confined area only, was not de-identified and thus violated the PDPA. It was also alleged that NHIB illegally converted the NHID data to a National Health Insurance Research Database ("NHIRD") for public access without prior informed consent.

After some deliberation the Administrative Supreme Court of Taiwan eventually sided with NHIB, holding that NHIB's proposed use, although without prior personal informed consent, was a justifiable use covered under PDPA's exemptions clause. The Court responded that said use is permissible because it was related to NHIB's

statutory mandate. The Court reasoned that even though the use of data was not de-identified, the anonymization measure applied by NHIB was sufficient to minimize risk of undue exposure. The Court also emphasized that a personal entitlement to privacy protection is not an absolute legal interest by nature, and may have to yield for comparatively bigger public interest when necessary.

Nonetheless, uncertainty remains after the Court's ruling. While human right groups continue questioning the legitimacy of academic access to NHID data, whether a for-profit organization, such as a pharmaceutical company, may ask for the similar access has yet to be clarified as well. Without these PDPA issues being resolved, it becomes challenging for Taiwan Biobank to develop a legally admissible cross database convergence scheme, so as to assist MOHW in complementing the BIIP goals.

## 3. The Opportunity: The Preliminary Broad Consent Arrangement of Taiwan Biobank Makes Possible the Cross Database Convergence

Taiwan Biobank is a national biobank created under MOHW's supervision, aiming to collect 200,000 healthy participants and 100,000 individuals with 12 specific diseases to form the largest population-based biobank in Taiwan [7]  As facilitation of cross database linkage has been its fundamental goal, every participant in Taiwan Biobank has been informed and consented during the recruitment process, including for any future cross database linkage, and including for those personal data collected in NHID. Theoretically, the informed consent obtained by Taiwan Biobank might have satisfied the exemption clause stipulated under Sub. Para. 6 of Para. 1 of Art.6 of PDPA (Table. 1). The consent obtained during Taiwan Biobank's recruitment may

already fit into the "prior personal consent with autonomy" exemption.

Table 1: Article 6 of Persona Data Protection Act ("PDPA")

Personal information like medical records, medical treatment, genetic information, sexual life, health examination and criminal records, etc., is sensitive in nature. Article 6 of PDPA provides that such information, in general, shall not be collected, processed, or used. Article 6 of PDPA enlists six exceptions to this restriction.

| Article 6 of PDPA: |
| --- |
| Personal information of medical records, medical treatment, genetic information, sexual life, health examination and criminal records should not be collected, processed or used. However, the following situations are not subject to the limits set in the preceding sentence: |
| 1. when in accordance with law; |
| 2. when it is necessary for a government agency to perform its legal duties or for a non- government agency to fulfill its legal obligation, and proper security measures are adopted prior or subsequent to such collection, processing or use; |
| 3. when the Party has made public such information by himself, or when the information concerned has been publicized legally; |
| 4. where it is necessary to perform statistical or other academic research, a government agency or an academic research institution collects, processes, or uses personal information for the purpose of medical treatment, public health, or crime prevention. The information may not lead to the identification of a specific person after its processing by the provider, or from the disclosure by the collector; |
| 5. where it is necessary to assist a government agency in performing its legal duties or a non-government agency in fulfilling its legal obligations, and proper security measures are adopted prior or subsequent to such collection, processing, or use; |
| 6. where the Party has consented in writing; unless such consent exceeds the necessary scope of the specific purpose; the collection, processing or use merely with the consent of the Party is prohibited by other statutes; or such consent is against the Party's will. |

Certainly, the informed consent by itself may not be deemed as a substitute of full Ethical, Legal & Social Implication (ELSI) compliance. This is especially true when the informed consent obtained by Taiwan Biobank has been a "broad" one; and the consent has been "for future research" in a general sense. Besides, to facilitate the use of biobank, the collected tissues and data need not be de-linked forever. Thus, subject to Taiwan's Human Biobank Management Act (HBMA) in 2010, the participants have been asked to grant Taiwan Biobank the privilege to maintain the "irretrievability" of

related data, under the governance of the ethical code of “re-contact“ and Ethical Governance Committee‘s (“EGC”) continuous supervision. Furthermore, Taiwan Biobank may not release its collection without EGC’s approval. Under this enhanced governance framework, more than 80,000 participants have been recruited to date without query against the legitimacy of Taiwan Biobank’s practices.

However, it was argued that not until the revision of PDPA, PDPA’s restriction shall be applicable in governing Taiwan Biobank‘s broad consent practice. While this article believes that such an interpretation contradicts the altruism and autonomy of participants, still, the social legitimacy concern laid behind the argument may not be ignored. Therefore, these authors suggested Taiwan Biobank may adopt an additional hash function to protect participants’ privacy. Hash function has been adopted in biobank such as Vanderbilt DNA databank [8], and has proved to be useful in linking DNA data with health data in de-identified fashion. By replacing the participant’s ID with a Hash value returned by a hash function, it is further ensured that participant’s identity cannot be regenerated from the same hash output (Figure 2).

With the sound broad consent from the participant for possible linkage, the ongoing supervision of EGC, and an additional hash function to bridge the gap between HBMA and PDPA, it becomes possible for the Taiwan Biobank, when trying to link with NHID, to survive the strictest scrutiny of patient and human right groups concerning PDPA compliance.

## 4. Conclusion:

In the Big Data era, precision medicine is not achievable without cross database convergence in various settings among genetic, environmental and EMR. Nonetheless, the rigid protection of privacy has become great obstacle for biobank to access health database. This article believes a consent-based approach will help ease up the concern over violation of PDPA, and clear up the path for making NHID accessible for research purpose. Further, with more and more national biobanks like PMI-CP and UK Biobank established, the need for establishing a unified regional or international standard to make sure interoperability of EMR will be on the rise. When implementing BIIP, MOHW may use Taiwan Biobank as a role model to standardize the procedure for access of EMR in Taiwan, and pave the way for Taiwan to play a more active role in the global biobank network.

**Reference**


1. Consortium IHGS: **Finishing the euchromatic sequence of the human genome**. *Nature* 2004, **431**(7011):931-945.
2. Wu T-Y, Majeed A, Kuo KN: **An overview of the healthcare system in Taiwan**. *London journal of primary care* 2010, **3**(2):115-119.
3. Wei W-Q, Denny JC: **Extracting research-quality phenotypes from electronic health records to support precision medicine**. *Genome medicine* 2015, **7**(1):41.
4. Manolio TA, Abramowicz M, Al-Mulla F, Anderson W, Balling R, Berger AC, Bleyl S, Chakravarti A, Chantratita W, Chisholm RL: **Global implementation of genomic medicine: we are not alone**. *Science translational medicine* 2015, **7**(290):290ps213-290ps213.
5. Program CoBII: **Taiwan Biomedical Industry-Where Innovation Happens**. *http://wwwbiopharmorgtw/bio/2017/images/incentives/Taiwan%20Biomedic*

al%20Industry%20brochure%20(BIIP%20&%20BPIPO)pdf last viewed: 2017.09.15.

6. Chang C-H: **Controversy over Information Privacy Arising from the Taiwan National Health Insurance Database Examining the Taiwan Taipei High Administrative Court Judgment No. 102-Su-36 (TSAI v. NHIA)**. *Pace Int'l L Rev* 2016, **28**:29.

7. Chalmers D, Nicol D, Kaye J, Bell J, Campbell AV, Ho CW, Kato K, Minari J, Ho C-h, Mitchell C: **Has the biobank bubble burst? Withstanding the challenges for sustainable biobanking in the digital era**. *BMC medical ethics* 2016, **17**(1):39.

8. Roden DM, Pulley JM, Basford MA, Bernard GR, Clayton EW, Balser JR, Masys DR: **Development of a large-scale de-identified DNA biobank to enable personalized medicine**. *Clinical Pharmacology & Therapeutics* 2008, **84**(3):362-369.

## List of abbreviations

Biomedical Industry Innovation Program (BIIP)

Ethical Governance Committee (EGC)

Ethical, Legal & Social Implication (ELSI)

Human Biobank Management Act (HBMA)

Ministry of Health and Welfare (MOHW)

National Health Insurance Bureau (NHIB)

National Health Insurance Database (NHID)

National Health Insurance Research Database (NHIRD)

Personal Data Protection Act (PDPA)

## Ethics approval and consent to participate

**Consent for publication**

**Availability of data and material**

**Competing interests**

**None**

**Funding**

**None**

**Authors' contributions**

**Acknowledgements**

Table Click here to download Table Table 1 Article 6 of PDPA.doc

Table 1: Article 6 of Persona Data Protection Act ("PDPA")

Personal information like medical records, medical treatment, genetic information, sexual life, health examination and criminal records, etc., is sensitive in nature. Article 6 of PDPA provides that such information, in general, shall not be collected, processed, or used. Article 6 of PDPA enlists six exceptions to this restriction.

| |
|---|
| Article 6 of PDPA: |
| Personal information of medical records, medical treatment, genetic information, sexual life, health examination and criminal records should not be collected, processed or used. However, the following situations are not subject to the limits set in the preceding sentence: |
| 1. when in accordance with law; |
| 2. when it is necessary for a government agency to perform its legal duties or for a non- government agency to fulfill its legal obligation, and proper security measures are adopted prior or subsequent to such collection, processing or use; |
| 3. when the Party has made public such information by himself, or when the information concerned has been publicized legally; |
| 4. where it is necessary to perform statistical or other academic research, a government agency or an academic research institution collects, processes, or uses personal information for the purpose of medical treatment, public health, or crime prevention. The information may not lead to the identification of a specific person after its processing by the provider, or from the disclosure by the collector; |
| 5. where it is necessary to assist a government agency in performing its legal duties or a non-government agency in fulfilling its legal obligations, and proper security measures are adopted prior or subsequent to such collection, processing, or use; |
| 6. where the Party has consented in writing; unless such consent exceeds the necessary scope of the specific purpose; the collection, processing or use merely with the consent of the Party is prohibited by other statutes; or such consent is against the Party's will. |

Figure 1: Flowchart of BIIP

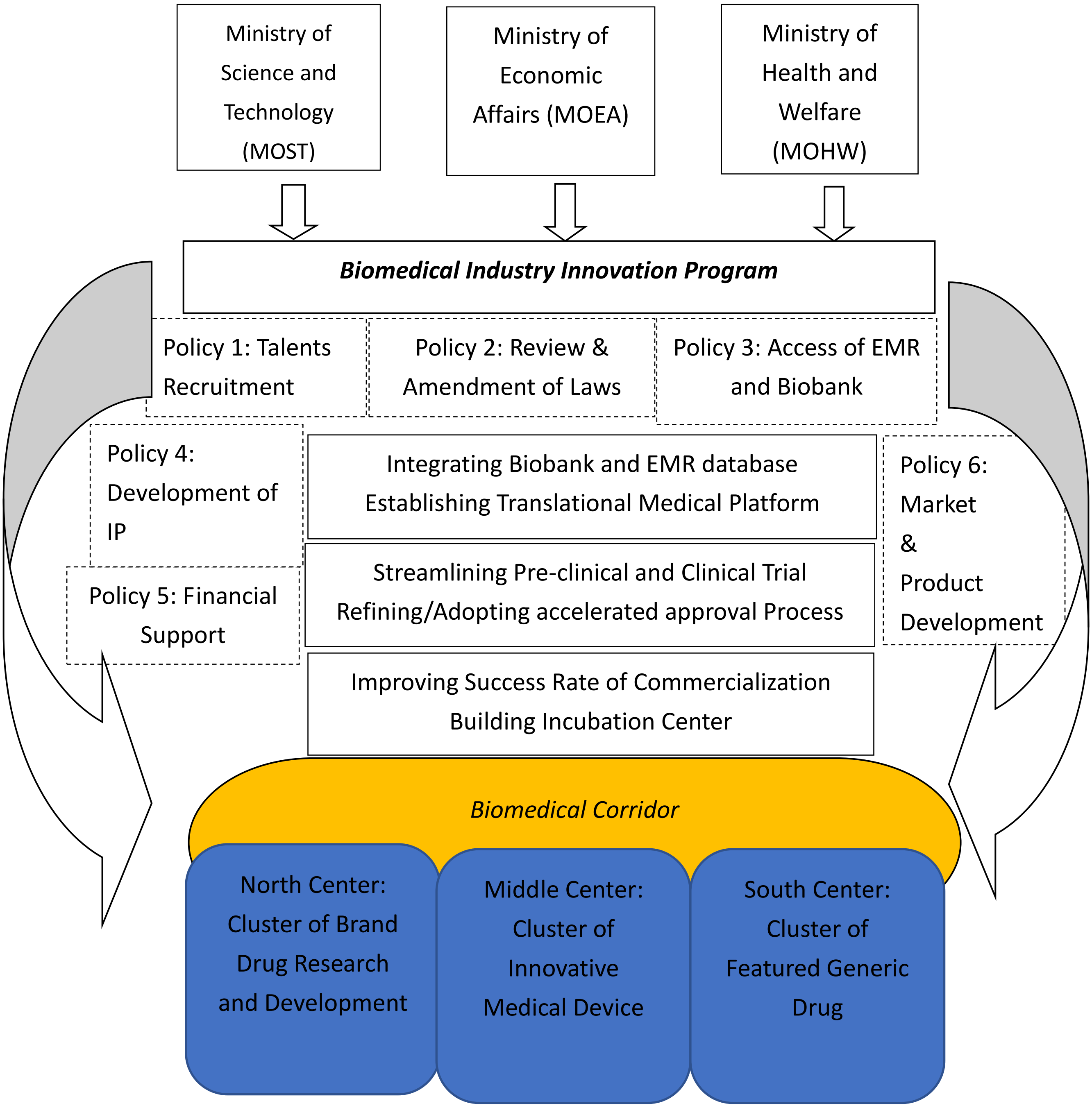


There are six featured policy aspects of BIIP, which is talents recruitment, amendment of law, increase access of EMR and Biobank, development of Intellectual Property right, providing financial support, and market and product development. By implementing these six policies, BIIP aims to establish a Biomedical Corridor that extends from north to south, with three major biomedical centers that will be the hubs for developing Taiwan's biomedical industry. Under such framework, the translational medical platform based on the integration of EMR and Biobank is the backbone for streamlining the biomedical research.

Figure
Click here to download Figure Figure 2 hashfunction framwork.doc

Figure 3: Flowchart of Hash function framework

One of the key features of hash function is that the algorithm will transform the identifiable personal data to a unique 128-character code. Taiwan Biobank will develop and enter into a protocol with NHID and adopt hash function framework. When there is need to access the EMR in NHID, all the identifiable data processed will be replaced by hash value returned by hash function, so the access of EMR will be proceeded in a de-identified manner.

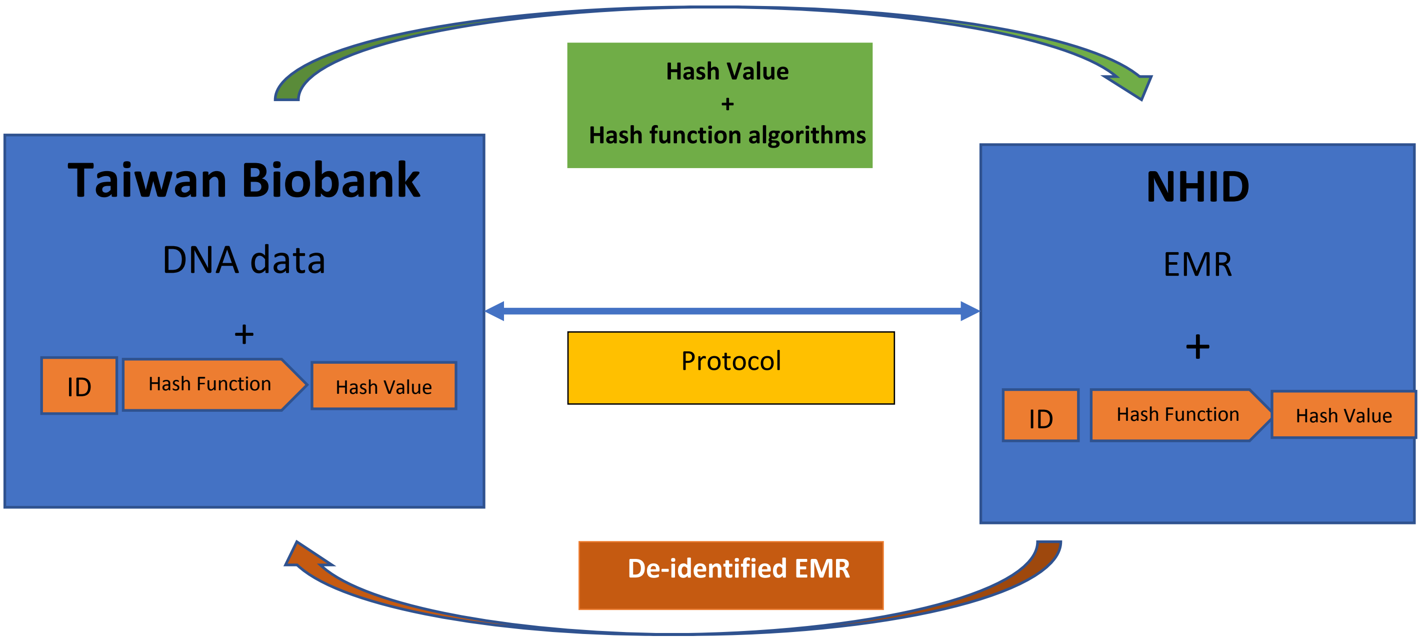

Supplement: GIGA-D-17-00249_Original-Submission.pdf [file gix110_giga-d-17-00249_original-submission.pdf]
