# Supplementary material for: Taiwan Biobank: making cross-database convergence possible in the Big Data era
Source: Gigascience. 2017 Nov 15;7(1):gix110. doi: 10.1093/gigascience/gix110 (PMC5774504; doi:10.1093/gigascience/gix110)
Supplement: Response-to-Reviewer-Comments_Original-Submission.pdf [file gix110_response-to-reviewer-comments_original-submission.pdf]

Author's Response To Reviewer Comments

All the editorial and reviewer's comments look fine to us. Modifications and/or adjustments have been completed accordingly.
