# Supplementary material for: Taiwan Biobank: making cross-database convergence possible in the Big Data era
Source: Gigascience. 2017 Nov 15;7(1):gix110. doi: 10.1093/gigascience/gix110 (PMC5774504; doi:10.1093/gigascience/gix110)
Supplement: Reviewer-1-Report-(Original-Submission) -- Ruth Stirton — 13 Oct 2017 Reviewed [file gix110_reviewer-1-report-(original-submission).pdf]

Reviewer Report

Title: Making Possible the Cross Database Convergence of Taiwan Biobank in Big Data Era

Version: Original Submission **Date:** 10/13/2017

Reviewer name: Ruth Stirton

Reviewer Comments to Author:

This is an interesting paper which highlights a specific legal difficulty in the Taiwan national biobank. Its focus is on the problem of consenting participants to have their tissue linked to their medical data and used in future research. There are significant potential privacy issues that people are worried about. The authors propose an additional level of anonymity and distancing between the person and their data, and that this will be sufficient to meet requirements of the law. I think that this is an interesting question which merits further discussion. Hopefully that discussion will be prompted by this commentary.I think the language, tone and content is of an appropriate standard for publication.

Level of Interest

Please indicate how interesting you found the manuscript: An article of importance in its field

Quality of Written English

Please indicate the quality of language in the manuscript: Acceptable

Declaration of Competing Interests

Please complete a declaration of competing interests, considering the following questions:

- Have you in the past five years received reimbursements, fees, funding, or salary from an organisation that may in any way gain or lose financially from the publication of this manuscript, either now or in the future?
- Do you hold any stocks or shares in an organisation that may in any way gain or lose financially from the publication of this manuscript, either now or in the future?
- Do you hold or are you currently applying for any patents relating to the content of the manuscript?
- Have you received reimbursements, fees, funding, or salary from an organization that holds or has applied for patents relating to the content of the manuscript?
- Do you have any other financial competing interests?
- Do you have any non-financial competing interests in relation to this paper?

If you can answer no to all of the above, write 'I declare that I have no competing interests' below. If your reply is yes to any, please give details below.

I declare that I have no competing interests.

I agree to the open peer review policy of the journal. I understand that my name will be included on my report to the authors and, if the manuscript is accepted for publication, my named report including any attachments I upload will be posted on the website along with the authors' responses. I agree for my report to be made available under an Open Access Creative Commons CC-BY license (http://creativecommons.org/licenses/by/4.0/). I understand that any comments which I do not wish to be included in my named report can be included as confidential comments to the editors, which will not be published.

I agree to the open peer review policy of the journal
